# Visual Search Task Reveals Categorical Representation of Topological Spatial Relations Between Objects

**DOI:** 10.5334/joc.523

**Published:** 2026-10-08

**Authors:** Jintao Song, Li Zheng, Zhentao Zuo, Tiangang Zhou, Yan Zhuo

**Affiliations:** 1 School of Education and Psychology, University of Jinan, Jinan, China; 2 State Key Laboratory of Cognitive Science and Mental Health, Institute of Biophysics, Chinese Academy of Sciences, Beijing, China; 3 University of Chinese Academy of Sciences, Chinese Academy of Sciences, Beijing, China; 4 Institute of Artificial Intelligence, Hefei Comprehensive National Science Center, Hefei, China; 5 National Key Laboratory of Human-Machine Hybrid Augmented Intelligence, Xi’an Jiaotong University, China

**Keywords:** topological structure, geometry, visual search task, spatial relations

## Abstract

Geometry provides a rigorous mathematical framework for understanding the spatial relations between objects. Topological properties are regarded as among the most fundamental invariants in geometry. To investigate how the visual system represents topological structures (e.g., inside/outside relations, connectivity), the current study employed the visual search task and visual stimulus sets designed by varying the distance between two objects equally. Results showed that task performance improved when the difference in object relations crossed category boundaries defined by topological properties. The study excluded the influence of potential confounding factors, such as the perceived depth caused by occlusion (experiments 1a and 1b). Furthermore, when the relevant inside/outside topological category boundary was removed, performance varied systematically with metric distance (experiment 1c). The categorical advantage was further observed across different modes of connectivity (experiments 2a, 2b, and 2c). These findings provided evidence for the categorical representation of topological spatial relations in visual perception.

## 1. Introduction

Our daily interactions involve interpreting relations between objects, a process central to visual perception (Hafri & Firestone, 2021). For instance, one can perceive how two puzzle pieces fit together or infer the original shape of a partially eaten cookie (Chen & Scholl, 2016; Hafri & Firestone, 2021). Additionally, a phone in a basket looks like a knife in a cup due to the same “in” relation (i.e., they are perceived as similar at the relational level because visual processing represents relations abstractly), despite the two images involving completely different objects (Hafri et al., 2024). The visual system intuitively abstracts network topological relations, like T-junctions, crosses, and holes in subway maps, enabling efficient navigation from one place to another (Yousif & Brannon, 2024, 2025). The aforementioned research has focused on the spatial relations between objects, suggesting that relational structures play a fundamental role in our understanding of the world.

One traditional approach to formalizing relational representations is through geometry. Various geometric categories have been investigated within the field of cognitive science, including basic shapes (e.g., square, rectangle, circle), Euclidean geometric properties (e.g., point, line, parallelism, angle, and symmetry), and topological properties (e.g., object topology: the number of holes, inside/outside relations, and connectivity; network topology: n-point vertex) (Chen, 1982; Chen, 2005, 2025; Elder, 2018; Elder et al., 2018; Elder & Zucker, 1993, 1994; Huttenlocher et al., 1991; Sablé-Meyer et al., 2022; Sablé-Meyer et al., 2021). The mathematician Klein advocated for an invariant perspective in considering geometric properties, emphasizing a structured hierarchy among geometries (Klein, 1893). In the domain of human form perception, Chen proposed a global-to-local functional hierarchy (i.e., ascending order of stability: Euclidean geometry, affine geometry, projective geometry, and topology) (Chen, 2005). These geometric categories possess varying degrees of stability, leading to differences in constraints when describing spatial relations between objects.

Some studies have discussed the relationship between topological structures and categorical representation. Lovett and Franconeri manipulated the distance between objects to establish topological relations, which they named touching, overlapping, and containing. Utilizing the sequence comparison task, they observed improved accuracy when these topological differences were present (Lovett & Forbus, 2009; Lovett & Franconeri, 2017). Burlinson and colleagues investigated closure in several experimental tasks, including the flanker, same-different reaction time, and visual analytic tasks. Their findings affirmed that closure can be recognized as a new perceptual category in visual displays (Burlinson, 2019; Burlinson et al., 2018). However, these tasks included multiple processing stages (i.e., encoding, retrieval, and comparison), making it impossible to isolate the stage responsible for the categorical effects. Moreover, they did not adequately control for the nontopological features of visual stimuli (e.g., area, the number of terminators), leaving the possibility that these features contributed to the observed effects. Therefore, a perception paradigm specifically designed to explore topological relations between objects was needed.

The visual search task is widely recognized as an effective methodological tool for investigating perceptual relations between objects (Hafri & Firestone, 2021; Wolfe, 2021). By presenting visual stimuli simultaneously, the visual search task avoids the confounds associated with multiple processing stage tasks. Furthermore, this task can be combined with precisely controlled stimuli to rule out alternative explanations. As demonstrated by Duncan and Humphreys, attention is more effectively guided to a target as the target-distractor (TD) difference increases (Duncan & Humphreys, 1989). Topological relations (e.g., inside/outside relation) indicate that an object is either inside or outside another, with little middle ground (Hafri & Firestone, 2021). This categorical nature of topological representation increases the TD difference, thereby enhancing search efficiency. Consequently, the visual search task is suitable for studying the representation of topological relations.

The current study employed the visual search task across six behavioral experiments to investigate whether topological relations between objects are represented categorically. Specifically, experiment 1 (1a, 1b, and 1c) examined inside/outside relations, whereas experiment 2 (2a, 2b, and 2c) focused on connectivity. If topological properties are represented as categorical variables, task performance should be improved when the stimuli crossed the category boundaries defined by the topological properties. In contrast, if the topological properties are represented as metric variables, the performance should be consistent across the visual stimulus sets because the distances between stimuli are equated.

## 2. General methods

### 2.1 Participants

A total of 116 participants took part in this study. All participants were right-handed and reported normal or corrected vision. Participants were recruited as follows: experiment 1a (N = 20; 7 males, 13 females; age range: 21–30 years, M ± SD = 23.65 ± 2.08), experiment 1b (N = 20; 3 males, 17 females; age range: 21–27 years, M ± SD = 23.50 ± 1.61), experiment 1c (N = 20; 7 males, 13 females; age range: 18–31 years, M ± SD = 23.30 ± 2.62), experiment 2a (N = 20; 9 males, 11 females; age range: 19–30 years, M ± SD = 23.75 ± 2.65), experiment 2b (N = 18; 5 males, 13 females; age range: 19–26 years, M ± SD = 22.94 ± 1.89), experiment 2c (N = 18; 3 males, 15 females; age range: 18–25 years, M ± SD = 22.61 ± 1.91). The sample size was determined using G*Power analysis (Faul et al., 2009). The assumed effect size was based on effects reported in previous topological discrimination tasks (Chen, 2005). The power analysis indicated that a sample size of 15 participants yields a power of 0.8 to detect the effects with an effect size of 0.3. All participants provided informed consent and received compensation after completing the task.

### 2.2 Visual stimuli

The parameters of the visual stimuli (Figure 1) in the experiments were as follows:

**Figure 1:**
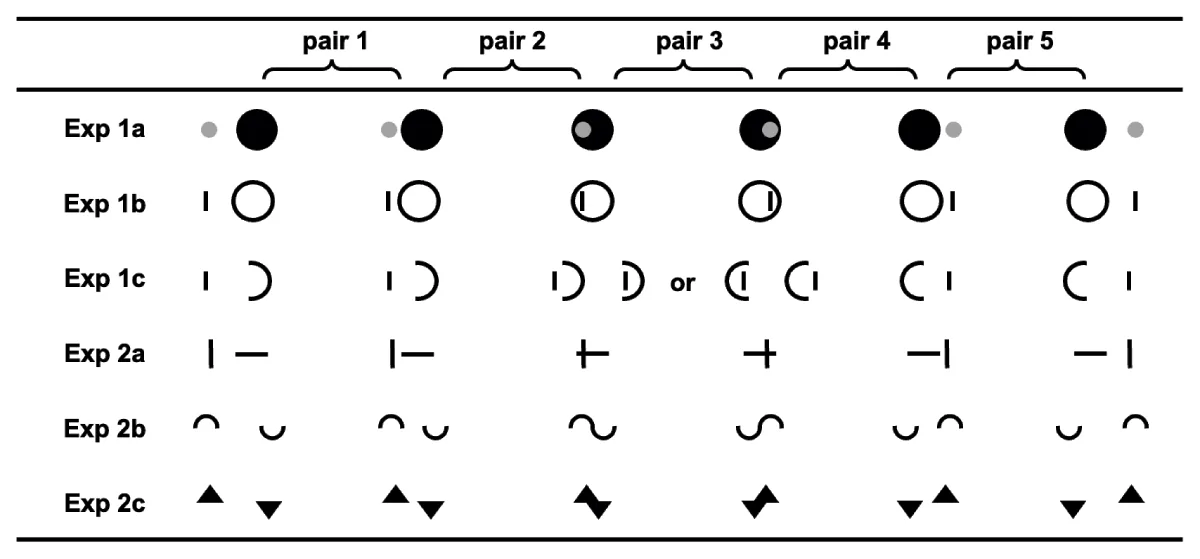
**Stimulus Sets**. Each row shows the stimuli of one experiment, and any two adjacent columns form a stimulus pair. Exp 1a, 1b, 2a, 2b, and 2c: Pairs 1, 3, and 5 differed in relative distance, not in inside/outside relation or connectedness. Pairs 2 and 4 differed in topological properties (i.e., inside vs. outside, connected vs. unconnected). (i.e., Exp 1a: The small circles were white in the experiment, presented in gray for display reasons here). Exp 1c: The inside/outside topological category boundary was removed.

- Experiment 1a: The radius of the small white circle was 0.4° (RGB: 255, 255, 255), and the radius of the large black circle was 1° (RGB: 0, 0, 0). The small white circle was shifted by –1°, –0.6°, –0.2°, 0.2°, 0.6°, and 1° from the large black circle’s center.
- Experiment 1b: The stimuli consisted of two objects: a bar and a circle. The viewing angle of the width and length of the bar were 0.12° and 0.6°, while the thickness and radius of the circle were 0.1° and 1°. The bar’s location was –1°, –0.6°, –0.2°, 0.2°, 0.6°, and 1° from the center of the circle.
- Experiment 1c: Stimuli 1–3 had their arc openings facing left, whereas stimuli 4–6 had their openings facing right. For target-present trials involving pair 3, one stimulus was horizontally reversed so that the target and distractors had the same orientation; displays could therefore be either all left-facing or all right-facing.
- Experiment 2a: The stimuli consisted of two bars: the length was 2°, and the width was 0.12°. The vertical bar was positioned at –2.5°, –1.5°, –0.5°, 0.5°, 1.5°, and 2.5° from the center of the horizontal bar, horizontally.
- Experiment 2b: The radius of the black arc was adjusted to 0.25°, and the distance from the initial position to the center of the screen was 8°. The horizontal distances from the center of the upper arc to the lower arc were –2.5°, –1.5°, –0.5°, 0.5°, 1.5°, and 2.5°.
- Experiment 2c: The base and height of the triangle were 2° and 1°. The horizontal distances between the upper triangle and the lower triangle were –2°, –1.2°, –0.4°, 0.4°, 1.2°, and 2°.

### 2.3 Procedure

The visual stimuli were presented on a 24-inch LCD screen through the Psychtoolbox (Brainard, 1997; Pelli, 1997) based on MATLAB (The MathWorks, Inc., Natick, MA, USA), with a resolution of 1920 * 1080 and a refresh rate of 60 Hz. Participants sat approximately 70 cm away from the screen and were required to respond as quickly as possible without sacrificing accuracy.

The visual stimuli were presented on a gray background and positioned 7° from the center of the screen, horizontally and vertically. Subsequently, random jitter ranging from –2° to 2° was applied in horizontal and vertical directions independently. The disturbance was equal to the circle and bar to ensure the relative positions remained unchanged. Each trial began with a fixation lasting 200 ~ 400 ms. Then, visual stimuli appeared simultaneously in the four quadrants until the participants responded (Figure 2). The task required participants to report whether one quadrant differed from the rest or not. An auditory warning signal was triggered after the incorrect responses. The interval between trials was 500 ms.

**Figure 2:**
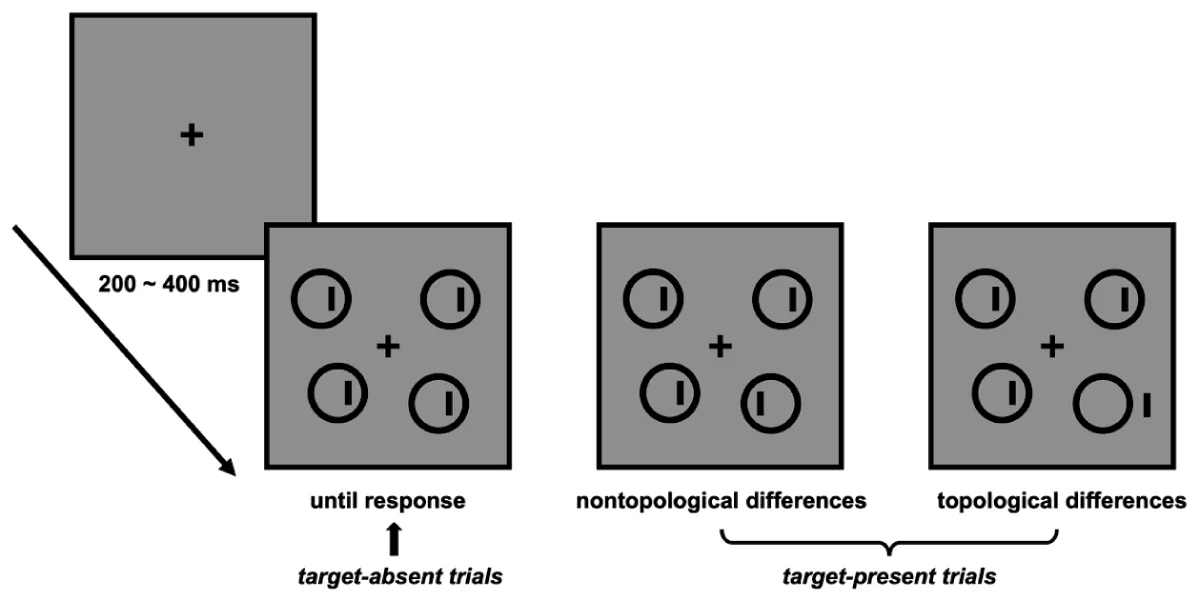
**Experiment task flow**. In the trial flow (*target-absent trials*), participants were expected to press the identical button as the visual stimuli in the four quadrants were the same. In the two trials on the right (*target-present trials*), participants were expected to press the different button as the stimulus in one quadrant differed from the rest: local geometric differences (all inside relations or all outside relations) and topological property differences (difference in inside/outside relations). The visual stimuli do not appear aligned in the square array due to the random jitter in the experiments.

The 480 trials were classified into two types based on the correct response: *target-absent trials* and *target-present trials*. In the *target-absent trials*, the correct response was ‘same’ because identical stimuli were presented in four quadrants. Each of the six stimuli was presented 40 times, resulting in a total of 240 trials. In the *target-present trials*, the correct response was ‘different’ because the stimulus presented in one quadrant differed from the rest. In the visual stimulus sets, any two adjacent stimuli constituted a pair, resulting in five stimulus pairs (Figure 1, from left to right, stimulus pair 1 to 5). Stimulus pairs 2 and 4 were topologically different (e.g., difference in connectivity, or inside/outside relations), while pairs 1, 3, and 5 were topologically equivalent but differed in other geometric features (e.g., difference in distances between objects). To balance the number of topological and local geometric differences in *target-present trials* (30 trials each for pairs 2 and 4, and 20 trials each for pairs 1, 3, and 5), resulting in another 240 trials. Different types of trials were randomly presented during the experiment. There were practice trials before the formal experiment to ensure participants were familiar with the task. The appropriate Ethics Committee approved the experiments.

### 2.4 Data analysis

The perceptual similarity of stimuli was operationally defined as the average reaction time between two adjacent stimuli. One stimulus pair consisted of two types of trials. In the first type of trial, the left stimulus was the target and the right stimulus was the distractor. In the second type of trial, the right stimulus was the target and the left stimulus was the distractor. Three distractors and one target were presented in the *target-present trials*. The statistical analysis of accuracy rate and reaction time was focused on the *target-present trials*. Only correct trials with reaction times between 200 and 2000 ms were included, and those exceeding 2.5 standard deviations from the mean were further excluded. The data analysis focused on adjacent stimulus pairs, as the *target-present trial* contained only a single stimulus pair. Greenhouse-Geisser correction was applied to adjust the *p*-values, and the Bonferroni correction was used to compare conditions.

## 3. Results

### 3.1 Experiment 1a: Inside/outside relations

According to the topological perception theory, the inside/outside relations are topological invariants that remain unchanged over shape-changing transformations (Chen, 1982; Chen, 2005). Experiment 1a used a large black circle and a small white circle as the visual stimuli, which had been previously used in Lovett and Franconeri’s study. The experiment aimed to validate the categorical representation of topological spatial relations using the visual search task.

The reaction times for each stimulus pair on *target-present trials* were as follows: pair 1 (1155 ± 178 ms), pair 2 (940 ± 156 ms), pair 3 (1166 ± 210 ms), pair 4 (930 ± 151 ms), and pair 5 (1102 ± 167 ms). The reaction times were analyzed through the one-way repeated measures ANOVA for 5 stimulus pairs (pair 1, pair 2, pair 3, pair 4, pair 5) (Figure 3a). The main effect of the stimulus pair was significant, *F* (4, 76) = 53.703, *p* < 0.001, $\eta_{p}^{2}$ = 0.739. Bonferroni-corrected multiple-comparison post hoc tests showed significant differences between adjacent stimulus pairs: pairs 1 and 2 (*t* = 10.651, *p* < 0.001, Cohen’s d = 1.236, 95% CI = [150.538, 278.333]), pairs 2 and 3 (*t* = –7.499, *p* < 0.001, Cohen’s d = –1.299, 95% CI = [–320.774, –130.006]), pairs 3 and 4 (*t* = 7.831, *p* < 0.001, Cohen’s d = 1.359, 95% CI = [140.216, 331.314]), pairs 4 and 5 (*t* = –10.201, *p* < 0.001, Cohen’s d = –0.989, 95% CI = [–225.036, –118.239]).

**Figure 3:**
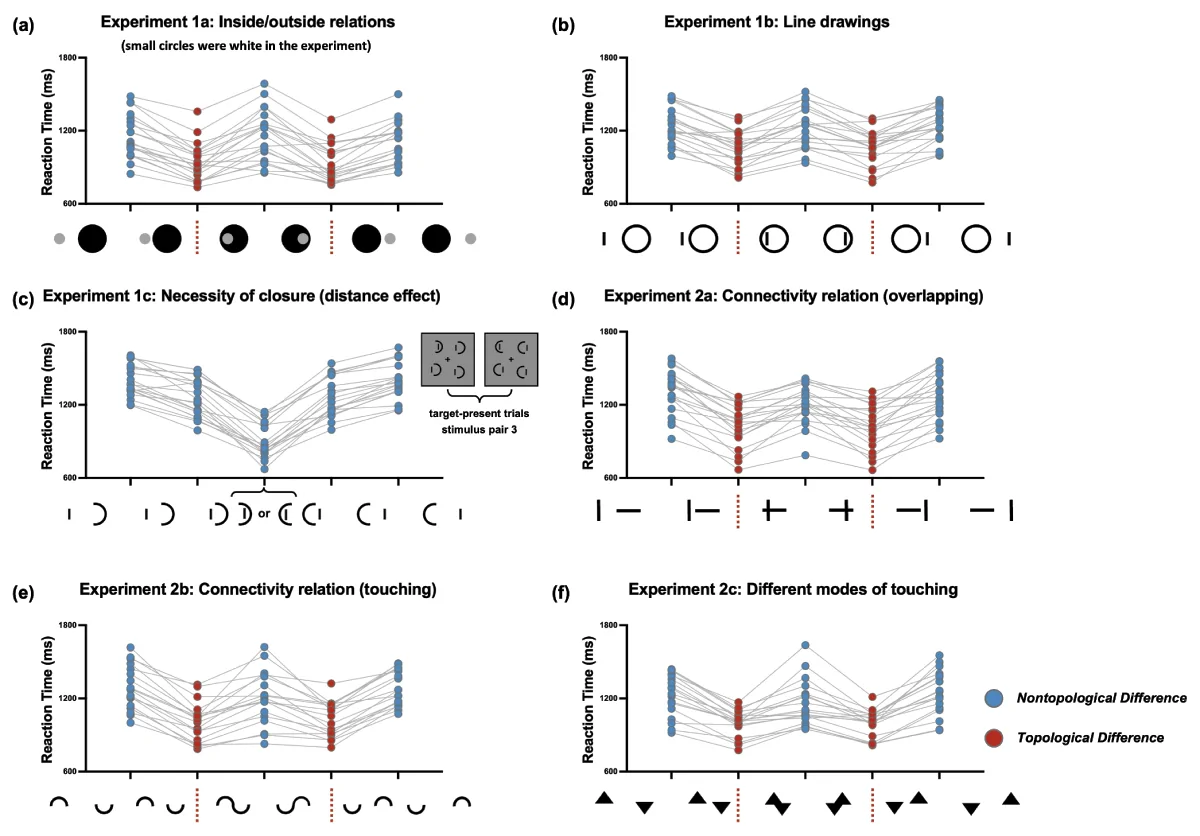
**Results from experiments. Exp 1a, 1b, 1c, 2a, 2b and 2c, panels a-f**. **(a)** Exp 1a: A small white circle and a large black circle were adopted to test inside/outside relations. The small circles were white in the experiment, presented in gray for display reasons here. **(b)** Exp 1b: A closed circle and a bar were adopted to exclude the influence of occlusion in Exp 1a. **(c)** Exp 1c: The inside/outside topological category boundary was removed. **(d)** Exp 2a: Two bars were adopted to test connectivity defined by overlapping. **(e)** Exp2b: Two arc stimuli were adopted to test connectivity defined by touching. **(f)** Exp 2c: Two triangles were adopted to examine connectivity with a larger touching area. Exp 1a, 1b, 2a, 2b, and 2c: Most participants showed a W-shape pattern between different conditions, with faster response times for topological differences (red dots) than for nontopological differences (blue dots). Exp 1c: Most participants showed a V-shape pattern between different conditions, and the response time increased with the distance between the two objects (blue dots). Each dot represents the results of one participant.

The total accuracy rate in Experiment 1a was 95.65% (*SD* = 2.80%). The accuracy rates for each stimulus pair were as follows: pair 1 (92.75 ± 5.73%), pair 2 (98.00 ± 2.83%), pair 3 (96.60 ± 3.12%), pair 4 (98.55 ± 1.90%), pair 5 (90.30 ±7.02%). We employed the same design to assess accuracy rate and observed that the difference between stimulus pairs was either nonsignificant (pairs 2 and 3, pairs 3 and 4, *Ps* > 0.068) or consistent with the reaction times (i.e., slower responses were associated with lower accuracy, pairs 1 and 2, pairs 4 and 5, *Ps* <0.005). Therefore, there was no evidence of a speed-accuracy trade-off. The results showed that performance improved when the difference crossed the inside/outside relations boundaries (i.e., an inside target among the outside distractors, an outside target among the inside distractors). Experiment 1a supported the categorical representation of topological relations between objects using the visual search task.

### 3.2 Experiment 1b: Line drawings

Beyond the perception of 2D topological structure, Experiment 1a involved depth perception (e.g., occlusion). Stimulus pairs 2 and 4 crossed both the boundaries of inside/outside relations and depth cues (i.e., inside relations involve occlusion, outside relations do not involve occlusion). The perceived depth resulting from occlusion might have influenced the results because occluded shapes are perceived relatively farther away (Yousif & Scholl, 2019). Experiment 1b employed line drawings (Chen, 1982; Wang et al., 2007) as the visual stimuli to investigate inside/outside relations without the confusion of depth or occlusion.

The reaction times for each stimulus pair on *target-present trials* were as follows: pair 1 (1246 ± 146 ms), pair 2 (1056 ± 143 ms), pair 3 (1230 ± 171 ms), pair 4 (1071 ± 149 ms), and pair 5 (1249 ± 142 ms). The reaction times were analyzed through the same ANOVA for stimulus pairs (Figure 3b). The main effect of the stimulus pair was significant, *F* (4, 76) = 47.308, *p* < 0.001, $\eta_{p}^{2}$ = 0.713. Bonferroni-corrected multiple-comparison post hoc tests showed significant differences between adjacent stimulus pairs: pairs 1 and 2 (*t* = 9.757, *p* < 0.001, Cohen’s d = 1.257, 95% CI = [127.944, 251.302]), pairs 2 and 3 (*t* = –8.300, *p* < 0.001, Cohen’s d = –1.150, 95% CI = [–239.763, –107.120]), pairs 3 and 4 (*t* = 7.274, *p* < 0.001, Cohen’s d = 1.053, 95% CI = [89.493, 228.040]), pairs 4 and 5 (*t* = –8.260, *p* < 0.001, Cohen’s d = –1.182, 95% CI = [–246.783, –109.781]).

The total accuracy rate in Experiment 1b was 92.95% (*SD* = 3.49%). The accuracy rates for each stimulus pair were as follows: pair 1 (86.85 ± 7.30%), pair 2 (96.80 ± 3.40%), pair 3 (94.60 ± 4.31%), pair 4 (97.75 ± 2.31%), pair 5 (85.05 ± 9.47%). We employed the same design to assess accuracy rate and observed that the difference between stimulus pairs was either nonsignificant (pairs 2 and 3, *p* = 0.778) or consistent with the reaction times (i.e., slower responses were associated with lower accuracy, pairs 1 and 2, pairs 3 and 4, pairs 4 and 5, *Ps* <0.039). Therefore, there was no evidence of a speed-accuracy trade-off. The results showed that performance improved when the difference crossed the inside/outside relations boundaries, defined by a closed circle and a bar. Experiment 1b suggested that the inside/outside relations were decisive factors in the categorical representation rather than depth perception caused by occlusion.

### 3.3 Experiment 1c: Necessity of closure (distance effect)

The inside/outside relations depend on a closed boundary, and the effects of other factors remain unclear when the closure is disrupted. On the other hand, the visual stimuli in Experiments 1a and 1b were designed by manipulating the distance between the objects, which raises the question of whether the observed results could be attributed to the manipulation. To address this concern, Experiment 1c employed stimulus pairs consisting of one straight line and one arc to disrupt the enclosing contour. This manipulation removed the relevant inside/outside topological category boundary while preserving the original metric distance differences between the objects across stimulus pairs.

The reaction times for each stimulus pair on *target-present trials* were as follows: pair 1 (1399 ± 130 ms), pair 2 (1249 ± 149 ms), pair 3 (909 ± 141 ms), pair 4 (1260 ± 155 ms), and pair 5 (1401 ± 145 ms). The reaction times were analyzed through the same ANOVA for stimulus pairs (Figure 3c). The main effect of the stimulus pair was significant, *F* (4, 76) = 159.292, *p* < 0.001, $\eta_{p}^{2}$ = 0.893. Bonferroni-corrected multiple-comparison post hoc tests showed significant differences between adjacent stimulus pairs: pairs 1 and 2 (*t* = 6.918, *p* < 0.001, Cohen’s d = 1.047, 95% CI = [81.714, 220.235]), pairs 2 and 3 (*t* = 14.364, *p* < 0.001, Cohen’s d = 2.358, 95% CI = [264.880, 415.134]), pairs 3 and 4 (*t* = –15.154, *p* < 0.001, Cohen’s d = –2.438, 95% CI = [–425.067, –277.849]), pairs 4 and 5 (*t* = –11.039, *p* < 0.001, Cohen’s d = –0.974, 95% CI = [–180.857, –100.089]).

The total accuracy rate in Experiment 1c was 90.40% (*SD* = 5.55%). The accuracy rates for each stimulus pair were as follows: pair 1 (81.40 ± 14.26%), pair 2 (93.25 ± 6.28%), pair 3 (99.10 ± 1.62%), pair 4 (93.65 ± 4.52%), pair 5 (81.65 ± 10.50%). We employed the same design to assess accuracy rate and observed that the difference between stimulus pairs was consistent with the reaction times (i.e., slower responses were associated with lower accuracy, pairs 1 and 2, pairs 2 and 3, pairs 3 and 4, pairs 4 and 5, *Ps* <0.008). Therefore, there was no evidence of a speed-accuracy trade-off. By calculating the difference scores between transitions (i.e., difference 1: pair 1–2, difference 2: pair 2–3, difference 3: pair 4–3, difference 4: pair 5–4), we found that the effect was indeed more dramatic for transitions to and from pair 3: difference 1 and 2 (*t* = –5.225, *p* < 0.001), difference 2 and 3 (*t* = –0.862, *p* = 0.399), difference 3 and 4 (*t* = 7.498, *p* < 0.001). Notably, transitions to and from pair 3 (i.e., outside vs. partially contained) represent a relational change to which the visual system may be sensitive (Hafri & Firestone, 2021), rather than a “pure topology” effect. The findings showed that, after the relevant inside/outside topological category boundary was removed, performance varied systematically with metric distance, while other relational differences remained (e.g., transitions involving pair 3).

### 3.4 Experiment 2a: Connectivity relation (overlapping)

Experiment 2 investigated connectivity, another topological invariant (Chen, 2005). The connectivity was defined by spatial overlapping in experiment 2a, where two objects are connected if they share the same spatial area in a 2-dimensional view. In the experiment, two bar stimuli either overlapped to form a plus (connected condition) or remained spatially separated (disconnected condition).

The reaction times for each stimulus pair on *target-present trials* were as follows: pair 1 (1307 ± 180 ms), pair 2 (1031 ± 176 ms), pair 3 (1203 ± 155 ms), pair 4 (1025 ± 187 ms), and pair 5 (1266 ± 188 ms). The reaction times were analyzed through the same ANOVA for stimulus pairs (Figure 3d). The main effect of the stimulus pair was significant, *F* (4, 76) = 50.725, *p* < 0.001, $\eta_{p}^{2}$ = 0.728. Bonferroni-corrected multiple-comparison post hoc tests showed significant differences between adjacent stimulus pairs: pairs 1 and 2 (*t* = 10.771, *p* < 0.001, Cohen’s d = 1.555, 95% CI = [194.813, 357.589]), pairs 2 and 3 (*t* = –7.517, *p* < 0.001, Cohen’s d = –0.970, 95% CI = [–245.155, –99.594]), pairs 3 and 4 (*t* = 6.973, *p* < 0.001, Cohen’s d = 1.004, 95% CI = [97.150, 259.455]), pairs 4 and 5 (*t* = –8.186, *p* < 0.001, Cohen’s d = –1.354, 95% CI = [–333.817, –147.287]).

The total accuracy rate in Experiment 2a was 93.60% (*SD* = 5.17%). The accuracy rates for each stimulus pair were as follows: pair 1 (84.65 ± 14.54%), pair 2 (97.40 ± 2.16%), pair 3 (95.75 ± 4.44%), pair 4 (98.10 ± 1.68%), pair 5 (88.20 ± 8.91%). We employed the same design to assess accuracy rate and observed that the difference between stimulus pairs was either nonsignificant (pairs 2 and 3, *p* = 0.358) or consistent with the reaction times (i.e., slower responses were associated with lower accuracy, pairs 1 and 2, pairs 3 and 4, pairs 4 and 5, *Ps* <0.042). Therefore, there was no evidence of a speed-accuracy trade-off. The results showed that performance improved when the difference crossed the boundaries of connectivity defined by overlapping (i.e., a connected target among the unconnected distractors, an unconnected target among the connected distractors). The visual system categorically represented connectivity, consistent with the inside/outside relations.

### 3.5 Experiment 2b: Connectivity relation (touching)

Experiment 2b focused on the connectivity defined by touching. Two objects are connected if they are touching. The two arc stimuli either touched to form a horizontally S-shaped curve (connected condition) or remained spatially separated (unconnected condition).

The reaction times for each stimulus pair on *target-present trials* were as follows: pair 1 (1290 ± 180 ms), pair 2 (1005 ± 160 ms), pair 3 (1202 ± 218 ms), pair 4 (1024 ± 141 ms), and pair 5 (1278 ± 140 ms). The mean reaction times were analyzed through the same ANOVA for stimulus pairs (Figure 3e). The main effect of the stimulus pair was significant, *F* (4, 68) = 53.372, *p* < 0.001, $\eta_{p}^{2}$ = 0.758. Bonferroni-corrected multiple-comparison post hoc tests showed significant differences between adjacent stimulus pairs: pairs 1 and 2 (*t* = 12.293, *p* < 0.001, Cohen’s d = 1.677, 95% CI = [210.645, 360.315]), pairs 2 and 3 (*t* = –5.997, *p* < 0.001, Cohen’s d = –1.156, 95% CI = [–302.535, –91.036]), pairs 3 and 4 (*t* = 5.253, *p* < 0.001, Cohen’s d = 1.045, 95% CI = [68.766, 287.043]), pairs 4 and 5 (*t* = –12.140, *p* < 0.001, Cohen’s d = –1.491, 95% CI = [–321.166, –186.426]).

The total accuracy rate in Experiment 2b was 94.78% (*SD* = 2.56%). The accuracy rates for each stimulus pair were as follows: pair 1 (89.39 ± 7.78%), pair 2 (98.06 ± 1.89%), pair 3 (95.89 ± 3.38%), pair 4 (97.78 ± 1.63%), pair 5 (90.44 ± 5.69%). We employed the same design to assess accuracy rate and observed that the difference between stimulus pairs was either nonsignificant (pairs 2 and 3, pairs 3 and 4, *Ps* > 0.261) or consistent with the reaction times (i.e., slower responses were associated with lower accuracy, pairs 1 and 2, pairs 4 and 5, *Ps* <0.001). Therefore, there was no evidence of a speed-accuracy trade-off. The results showed that performance improved when the difference crossed the boundaries of connectivity defined by touching. The connectivity defined by touching was categorically represented as well.

### 3.6 Experiment 2c: Different modes of touching

Experiment 2c further examined whether the categorical connectivity advantage could also be observed with a different mode of touching involving a larger touching area. Two triangles were introduced, with the touching area at their bases being larger than that of the arc stimuli used in Experiment 2b.

The reaction times for each stimulus pair on *target-present trials* were as follows: pair 1 (1230 ± 173 ms), pair 2 (996 ± 108 ms), pair 3 (1162 ± 187 ms), pair 4 (992 ± 112 ms), and pair 5 (1247 ± 182 ms). The mean reaction times were analyzed through the same ANOVA for stimulus pairs (Figure 3f). The main effect of the stimulus pair was significant, *F* (4, 68) = 45.165, *p* < 0.001, $\eta_{p}^{2}$ = 0.727. Bonferroni-corrected multiple-comparison post hoc tests showed significant differences between adjacent stimulus pairs: pairs 1 and 2 (*t* = 9.766, *p* < 0.001, Cohen’s d = 1.493, 95% CI = [156.216, 310.069]), pairs 2 and 3 (*t* = –5.327, *p* < 0.001, Cohen’s d = –1.062, 95% CI = [–266.247, –65.548]), pairs 3 and 4 (*t* = 5.800, *p* < 0.001, Cohen’s d = 1.092, 95% CI = [75.780, 265.257]), pairs 4 and 5 (*t* = –9.035, *p* < 0.001, Cohen’s d = –1.636, 95% CI = [–346.624, –164.373]).

The total accuracy rate in Experiment 2c was 96.61% (*SD* = 1.69%). The accuracy rates for each stimulus pair were as follows: pair 1 (93.56 ± 4.69%), pair 2 (98.89 ± 1.81%), pair 3 (97.94 ± 2.58%), pair 4 (98.94 ± 1.11%), pair 5 (92.72 ± 5.28%). We employed the same design to assess accuracy rate and observed that the difference between stimulus pairs was either nonsignificant (pairs 2 and 3, pairs 3 and 4, *Ps* = 1.000) or consistent with the reaction times (i.e., slower responses were associated with lower accuracy, pairs 1 and 2, pairs 4 and 5, *Ps* <0.002). Therefore, there was no evidence of a speed-accuracy trade-off. The results showed that the categorical connectivity advantage was observed for both types of touching used in Experiments 2b and 2c, as evidenced by improved performance when the difference crossed the connectivity boundary.

## 4. Discussion

This study investigated the perception of spatial relations between objects using a topological approach. By combining the visual search task with the visual stimulus designed by varying the distance between two objects equally, the findings consistently revealed that task performance improved when the stimuli differed in the topological properties. Exp 1a utilized inside/outside stimulus pairs to establish the basic flow. Exp 1b adopted the line drawings to eliminate the potential influence of occlusion. Exp 1c introduced arc stimuli to investigate the necessity of closure and showed that performance varied systematically with metric distance after the relevant inside/outside topological category boundary was removed. Exp 2a focused on the connectivity defined by overlapping, and Exp 2b examined the connectivity defined by touching. Exp 2c further demonstrated that the categorical connectivity advantage was observed with a different mode of touching involving a larger touching area. In summary, the current study adopted the visual search task, which is well suited to revealing the visual system’s direct perception of abstract topological relations between objects.

### 4.1 The perception of topological relations

By demonstrating the visual system’s sensitivity to topological relations between objects, this study contributes to the growing field of relational perception. A core claim in this domain is that visual perception extracts not only individual object properties but also some relations between objects (Hafri & Firestone, 2021; Hafri & Papeo, 2025; Kaiser et al., 2019). At the level of individual objects, connectivity is a fundamental property that defines them as connected, bounded volumes with an inside and an outside. Connected entities constitute parts of a larger object (e.g., a bicycle’s wheels and frame), while disconnected entities form groups rather than objects (e.g., a flower arrangement or a flock of birds) (Cavanagh et al., 2023). At the level of visual scenes, relational representations between objects could also be formalized through geometric frameworks (e.g., physical relations such as *surround* involve inside/outside relations; *support, balance*, and *attach* embody the concept of connectivity; event relations like *intrude* and *deform* are shape-changing transformations). In summary, topological properties (e.g., connectivity, inside/outside relations) not only define individual perceptual objects but also the relations between objects.

Empirical evidence from vision science has revealed several critical characteristics of relational perception. First, relational structures influence scene interpretation. For instance, changes in containment or size relations between a person and a car can lead to different scene interpretations, such as vehicle breakdown, playing with a toy, or daily commuting (Summerfield et al., 2020). In the current experiments, elements presented within the four quadrants were identical in each trial. However, changing the topological relations between the elements had a significant impact on task performance. These results confirmed the critical role of some relational information in understanding visual scenes.

Secondly, the visual system spontaneously and rapidly represents the relations between objects. In exp 1a, participants irresistibly perceived the small white circle and the large black circle as having either inside or outside topological relations. Response times were significantly faster when the stimulus pairs differed in topological relations (i.e., stimulus pairs 2 and 4). The results aligned with previous findings using the brief exposure task, which found that topological properties could be extracted from exposures as short as 5 ms (Chen, 1982), suggesting the rapid processing of topological properties.

Furthermore, Kosslyn describes two distinct types of spatial relation representations in human vision: abstract categorical (i.e., the general properties of spatial structures by mapping configurations to broad equivalence classes) and specific coordinate spatial relations (i.e., indispensable for processing more precise information regarding the spatial location of objects) (Kosslyn et al., 1989). Topology focuses on properties (e.g., inside/outside relations, connectivity) that remain invariant over transformations, rather than the precise metric information (e.g., orientation, distance, and size). The current study found that task performance improved when the differences in relations crossed the boundaries of topological categories, providing evidence that topological relations were categorically represented (Burlinson, 2019; Lovett & Franconeri, 2017).

Finally, the perception of topological relations is a robust phenomenon. As illustrated by the task flow in target-present trials (Figure 2 *Target-present trials*), participants could more easily detect topological differences than nontopological differences in target-present trials. Additionally, the advantage effect was consistently observed across participants, with most showing improved performance when the stimuli varied in the topological properties. The present findings indicated that topological structures exhibit core characteristics of relational perception.

### 4.2 Functional hierarchy in human form perception

The design of visual stimuli in the current study was inspired by the seminal work of Lovett and Franconeri (2017), which manipulated the distance between objects to create different topological relations (Lovett & Franconeri, 2017). However, the current study differed from their work in several aspects. Lovett and Franconeri used the sequential same/different task, in which participants were required to report whether the first display differed from the second display. The first display was followed by a 1000 ms delay (i.e., 250 ms mask and 750 ms blank screen), which imposed high demands on participants’ short-term memory capacity. In contrast, the current study adopted the visual search task, which has been widely used in studies of the direct perception of relations between objects (Hafri & Firestone, 2021; Hafri & Papeo, 2025), such as physical instability (Yang & Wolfe, 2020), and social interactions (Papeo et al., 2019). Therefore, the present findings provide evidence for the robust representation of topological relations in visual perception.

Chen (2005) proposed a global-to-local functional hierarchy in human form perception. Consistent with this framework, the current experiments support a privileged role for topological properties in the representation of spatial relations (i.e., across six experiments, task performance was consistently modulated when the stimulus pairs crossed topological category boundaries, whereas distance effects emerged only after the relevant inside/outside topological category boundary was removed). Taken together, these findings indicate that whether a task involves changes in topological properties plays an important role in determining task performance. The priority of topological processing is further reflected in stimulus pair 3, which could also be interpreted as involving a symmetry relation (i.e., “left” vs. “right” within the large circle). However, the present results showed that reaction times for conditions involving symmetry relations (pair 3) were slower than those that differed in topological categories (pairs 2 and 4). In Lovett and Franconeri’s study, the relative priority or fundamentality of topological properties versus symmetry relations could not be distinguished (i.e., their results showed that accuracy for Interval 1 was comparable to categorical changes). The current findings, in turn, suggest a hierarchical account of human visual perception in which topological properties have a privileged role.

Meanwhile, the representation of shape (e.g., topology) reflects the core difference between cognition and computation (Chen, 2025). While numerous behavioral studies have demonstrated the importance and uniqueness of human shape perception (Chen, 1982; Chen, 2005; Elder, 2018; Elder et al., 2018; Elder & Zucker, 1993, 1994; Sablé-Meyer et al., 2022), computational models exhibit distinct representational patterns. For instance, Baker and Elder utilized animal silhouettes as visual stimuli and disrupted the object configuration while preserving local features. This manipulation influenced human task performance but did not impact deep convolutional neural networks (DCNNs), suggesting that neural networks are insensitive to object configuration (Baker & Elder, 2022). Similarly, Sablé-Meyer et al. used a geometric intruder task to examine how Euclidean geometric properties are represented. They found that a convolutional neural network for object recognition reflects baboons’ performance, but an additional symbolic model was needed to account for human performance (Sablé-Meyer et al., 2021). Consequently, the underlying mechanisms of shape representation distinguish human cognition from artificial neural networks.

### 4.3 Topological structures in visual perception

Experimental evidence on topological perception can be categorized into two distinct paradigms: *topological discrimination tasks* and *topological change detection tasks*. Topological discrimination tasks describe perceptual relations between objects. In this paradigm, figures with equivalent or different topological properties are presented simultaneously across different spatial locations. In contrast, the topological change detection tasks characterize the emergence of new perceptual objects through shape-changing transformations across frames. Specifically, changes in topological properties indicate the emergence of new perceptual objects, while the objects’ homogeneity is preserved over other geometric transformations (e.g., changes in orientation, distance, and size).

The visual search task used in the present study is a topological discrimination task. In *target-absent trials*, all stimuli were topologically equivalent; conversely, in *target-present trials*, the stimulus in one quadrant differed topologically from the others. Studies on topological discrimination often adopt the configural superiority effects, which emphasize that the structural relationships between components, rather than the components themselves, are more important in visual perception (Chen, 2005; Pomerantz et al., 1977). Compared with differences in individual line segments, differences in the topological structures formed by combining the line segments (e.g., the presence of a hole) facilitate discrimination (Chen, 2005). Moreover, some configural superiority effects previously reported (Eidels & Townsend, 2010; Pomerantz, 2003; Pomerantz & Pristach, 1989) may be attributed to topological structures, such as connectedness or closedness. These studies on topological discrimination provide a topological framework for characterizing perceptual relations between objects.

The topological change detection task involves shape-changing transformations. In the multiple objects tracking task, Zhou et al. found that compared with changes in other features or colors, alterations in topological properties during object motion impaired task performance, indicating that topological changes disrupt the representational continuity of perceptual objects (Zhou et al., 2010). Using a temporal order judgment task, Song et al. found that participants perceived topological changes earlier than nontopological changes. This finding supports the priority of topological changes in temporal order perception and provides evidence for attention capture by topological changes (Song et al., 2025). Furthermore, Wei et al. found a topological interference effect in a color visual working memory task. Although shape was a task-irrelevant dimension, the topological difference between the encoding and probe stages impaired color memory performance, suggesting that the visual system automatically encodes topological properties (Wei et al., 2021). In sum, these studies indicate that the visual system automatically prioritizes topological representations, highlighting their critical role in perceptual organization and attention capture.

### 4.4 Limitations

This study has several limitations. First, the present study focused on two types of topological properties (i.e., inside/outside relations and connectivity), but did not examine holes. Nevertheless, prior work has shown that holes are also categorically represented (Burlinson, 2019; Burlinson et al., 2018), thus the current findings provide complementary evidence for the categorical representation of topological properties. Second, a key strength of the present study lies in the stimulus design which manipulated topological relations by varying the distance between two objects equally. However, this design inevitably introduced potential influences of perceived object numerosity because topological properties play a foundational role in defining perceptual objects (He et al., 2009; He et al., 2015; Zhou et al., 2010). For example, in Exp 2a, stimuli 3 could be perceived as a single perceptual object, whereas stimuli 2 could be perceived as two perceptual objects. This limitation reflects a trade-off between maintaining equidistant changes in distance and controlling the number of perceptual objects (i.e., Yousif and Brannon held the number of objects constant while altering topological relations, which resulted in doubled distance differences, see Yousif & Brannon, 2025, Figure 2). Future studies could adopt alternative stimulus design strategies to disentangle changes in object numerosity from changes in topological relations. Third, the present study primarily focused on comparisons between adjacent stimulus pairs. This is because each *target-present trial* contained only a single stimulus pair, with strict control over distance manipulations. Future studies could design dedicated experiments focusing on more distant stimulus pairs to examine their differences in perceptual representations.

## 5. Conclusion

The current study investigated the perception of relations defined by two topological properties: inside/outside relations and connectivity. Using the visual search task, the results revealed that task performance improved when relational differences crossed category boundaries defined by these topological properties. Topological properties exhibit key characteristics of relational perception, including structural, categorical, and rapid processing. The results are consistent with a hierarchical account of human visual perception in which topological properties have a privileged role.

## Data Availability

All data and code for experiments are available at Open Science Framework https://osf.io/e4hrk/.
